# State Insane Asylum, Austin

**Published:** 1906-10

**Authors:** 


### STATE INSANE ASYLUM, AUSTIN.

Dr. B. M. Worsham, superintendent, in his report for 1905-6, shows the total number of patients for the fiscal year ending August 31, 1905, as 635 males, 533 women; total, 1172. For the year 1906 to August 31, 624 males, 547 women; total, 1171. Two hundred and fifteen were admitted; 232 were discharged, 137 died. Of the movement of population Dr. Worsham says:

"The movement of population has been slow because during the past four years the character of the cases admitted has not been favorable for much improvement. Many of the cases whose transcripts show that they have been insane for only a short period, turn out to be cases of high grade idiocy, or even worse, as far as any chance of permanent improvement of their mental condition is concerned. Such patients when admitted make up a large percentage of the standing population of the institution, their death being their only chance to leave it, and as this class of cases is as a rule exceedingly long lived, the percentage of moving population is necessarily cut down more and more each year. Of the 215 patients admitted during the year ending August 31, 1906, 113 were males and 102 females. Only eleven of this number were colored, five of whom were men and six women."

The report shows that of the deaths 36.2 per cent were from consumption; seven deaths from paresis, senility 6.

In his recommendations Dr. Worsham says that the law governing the admission of patients should be changed and modernized. "The plan of admission as it now stands," says Dr. Worsham, "on the statute books was arranged many years ago when conditions in this State were entirely different. The system is too expensive, and deficient in many ways. The court trials should be done away with, except when demanded by friends and relatives who question the advisability of the person being placed under restraint. A law similar in many respects to the one governing the admission into the epileptic colony would meet the requirements and be much less expensive to the different counties." Continuing Dr. Worsham says the law should be amended so that the State should be divided into three separate asylum districts, each district arranged in accordance with population.

Dr. Worsham also recommends, in addition to the regular appropriations, that the next Legislature appropriate $85,000 for one new building to accommodate 500 new patients, and one additional boiler and machinery to cost $10,000, and $10,000 for the erection of two new galleries on the female department. He also renews his recommendation, urging that some provision be made for the criminal insane of the State, so that they can be separated from other insane and safely kept. It is also an urgent demand that some provision be made for the defective children, idiots and imbeciles. They are still unprovided for, and something should be done for their relief.

The report of Superintendent Worsham on the Pasteur Institute shows that it has been a great success. During the first fiscal year 81 patients were treated and not a single case developed hydrophobia. The second year's operation of this department shows the total number of patients treated to be 254, 195 male and 59 female. The number of patients treated from Texas was 226, 20 from the Indian Territory, 4 from Oklahoma, 1 each from Arizona, Arkansas and Kansas and 1 from the Republic of Mexico. Out of the 254 treated there were only two deaths. The total fees collected from this department were $2410, and the disbursements were $1039.36. Speaking of this department, Dr. Worsham says:

"This department has grown so far beyond the expectations of everybody at the time it was created, I desire to call your attention to the fact that it requires almost the entire time of one assistant physician to attend to it. The work has so many exacting features connected with it, and the department is more than self-sustaining, I recommend that the man who does the detail part of the work should be paid an additional amount to the small salary appropriated for the assistant physician here, and that the amount be authorized paid out of the fees collected from non-indigent patients."

SOUTHWEST TEXAS INSANE ASYLUM, San Antonio, Texas, T. O. Maxwell, M. D., superintendent. Fifteenth annual report, year ending August 31, 1906: Remaining in asylum September 1, 1905, 806; admitted, 70; total, 876. Discharged, restored, 43; deaths, 23. On hand August 31, 1906, 735. Percentage of deaths, 2 6-10. On furlough, August 31, 1906, 55; escaped, 2; total, 792. Average daily population, 737. Daily cost of maintenance, per capita, 42 cents. The asylum is full to its capacity and more room is needed, for which Dr. Maxwell asks for an appropriation.

---

DOCTOR WANTED.—A fine opening for a doctor. Rich, black land; thickly settled; nice growing railroad town; practice established five years. Call on or write, DR. W. F. JOHNSTON, Otto, Texas, Falls county.

